# Mitigating saliva aerosol contamination in a dental school clinic

**DOI:** 10.1186/s12903-021-01417-2

**Published:** 2021-02-05

**Authors:** Maobin Yang, Asad Chaghtai, Marc Melendez, Hana Hasson, Eugene Whitaker, Mustafa Badi, Leona Sperrazza, Jeffrey Godel, Cemil Yesilsoy, Marisol Tellez, Santiago Orrego, Carolina Montoya, Amid Ismail

**Affiliations:** 1 Department of Endodontology, Maurice H Kornberg School of Dentistry, Temple University, Philadelphia, USA; 2 Environmental Health and Radiation Safety, Temple University Health Sciences Center, Philadelphia, USA; 3 Department of Restorative Dentistry, Maurice H Kornberg School of Dentistry, Temple University, Philadelphia, USA; 4 Department of Oral and Maxillofacial Pathology, Medicine and Surgery, Maurice H Kornberg School of Dentistry, Temple University, Philadelphia, USA; 5 Department of Orthodontics, Maurice H Kornberg School of Dentistry, Temple University, Philadelphia, USA; 6 Department of Oral Health Sciences, Maurice H Kornberg School of Dentistry, Temple University, Philadelphia, USA; 7 Maurice H Kornberg School of Dentistry, Temple University, Philadelphia, USA

**Keywords:** Aerosol, Dentistry, SARS‐CoV‐2, COVID-19, High-speed suction, Extra oral suction system

## Abstract

**Background:**

Transmission of COVID-19 via salivary aerosol particles generated when using handpieces or ultrasonic scalers is a major concern during the COVID-19 pandemic. The aim of this study was to assess the spread of dental aerosols on patients and dental providers during aerosol-generating dental procedures.

**Methods:**

This pilot study was conducted with one volunteer. A dental unit used at the dental school for general dental care was the site of the experiment. Before the study, three measurement meters (DustTrak 8534, PTrak 8525 and AeroTrak 9306) were used to measure the ambient distribution of particles in the ambient air surrounding the dental chair. The volunteer wore a bouffant, goggles, and shoe covers and was seated in the dental chair in supine position, and covered with a surgical drape. The dentist and dental assistant donned bouffant, goggles, face shields, N95 masks, surgical gowns and shoe covers. The simulation was conducted by using a high-speed handpiece with a diamond bur operating in the oral cavity for 6 min without touching the teeth. A new set of measurement was obtained while using an ultrasonic scaler to clean all teeth of the volunteer. For both aerosol generating procedures, the aerosol particles were measured with the use of saliva ejector (SE) and high-speed suction (HSS) followed a separate set of measurement with the additional use of an extra oral high-volume suction (HVS) unit that was placed close to the mouth to capture the aerosol in addition to SE and HSS. The distribution of the air particles, including the size and concentration of aerosols, was measured around the patient, dentist, dental assistant, 3 feet above the patient, and the floor.

**Results:**

Four locations were identified with elevated aerosol levels compared to the baseline, including the chest of the dentist, the chest of patient, the chest of assistant and 3 feet above the patient. The use of additional extra oral high volume suction reduced aerosol to or below the baseline level.

**Conclusions:**

The increase of the level of aerosol with size less than 10 µm was minimal during dental procedures when using SE and HSS. Use of HVS further reduced aerosol levels below the ambient levels.

## Background

Aerosols are solid or liquid particles generally smaller than 50 µm in diameter; while splatter are particles composed of a mixture of air, water and solid substances larger than 50 µm [1, 2]. Human daily physical activities, such as coughing, breathing, sneezing or laughing, produce bioaerosols. If bioaerosols contain pathogenic microorganisms such as bacteria or viruses, they become infectious and could be a major route of disease transmission. It is well established that aerosol particles of 10 µm or smaller pose the greatest health concern, as they are likely to remain airborne for a longer period and to enter the nasal passages and serve as carriers of respiratory diseases [3]. In addition, particles in the range of 10–20 µm may also evaporate, leaving droplet nuclei of contaminated material with a potential for viral transmission [4–6]. In the past, splatter and droplet nuclei have been implicated in the transmission of diseases such as SARS, measles and herpetic viruses [2].

Dental procedures using high-speed handpiece (HSH) or ultrasonic scalers (US) generate dental aerosols, which are produced by coolant/water in combination with compressed air and spraying. Dental aerosol is composed of various combinations of organic particles, saliva, blood or respiratory fluid, and may become contaminated with oral micro‐organisms [7]. The contamination from aerosol during dental procedures presents a potential significant hazard for the dental personnel, and universal precautions to limit aerosols should always be in place [2]. As regular saliva ejectors (SE) do not have the capacity to remove a significant amount of the aerosols and splatter [3], the American Dental Association (ADA) has recommended the use of high-speed suction (HSS) to minimize contaminated aerosols and splatter for infectious diseases. In recent years, several extraoral high volume suction systems (HVS), such as ADS, Tokyo Giken, AJAX have been introduced to the market. The ADS unit has a motor-driven high-power suction, and contains HEPA filtration system and a medical-grade UV-C light disinfectant system, which provide additional reduction of aerosol and disinfection of air in dental operatory.

The COVID-19 pandemic caused by SARS‐CoV‐2 has challenged the dental profession around the world because of the potential transmission by dental aerosol and splatter [8]. While dental splatters or saliva droplets usually fall due to gravity in an arch-like path, dental aerosols are capable of short- and long-range transport [4]. A previous study indicated that the microbiological contamination via aerosols was detected within 40 inches from oral cavity [9]. Since dentists and dental assistants usually operate at a distance of about 23 inches or less from a patient's oral cavity, the transmission of SARS-CoV-2 via aerosols is suggested in addition to transmission via droplets [10]. Other studies using bacteria culture methods have shown aerosol generating procedures produce a 15–30-fold increase in the number of colony-forming units cultivable from the air compared with pre-procedural levels [3], and can extend 1 to 4 feet from the field of operation [11, 12].

Currently, insufficient quantitative research is available regarding the particle size and concentration of dental aerosols that spread inside dental operatory. There is also a lack of studies that assess the effect of an extraoral suction system on eliminating/reducing dental aerosols. The aims of this proof-of concept study (n = 1) were to assess the size and concentration of dental aerosols that spread towards dental personnel and patients during dental procedures, and to evaluate the effectiveness of aerosol control using HSS + SE with or without HVS.

## Methods

### Dental procedures and positions

**Fig. 1:**
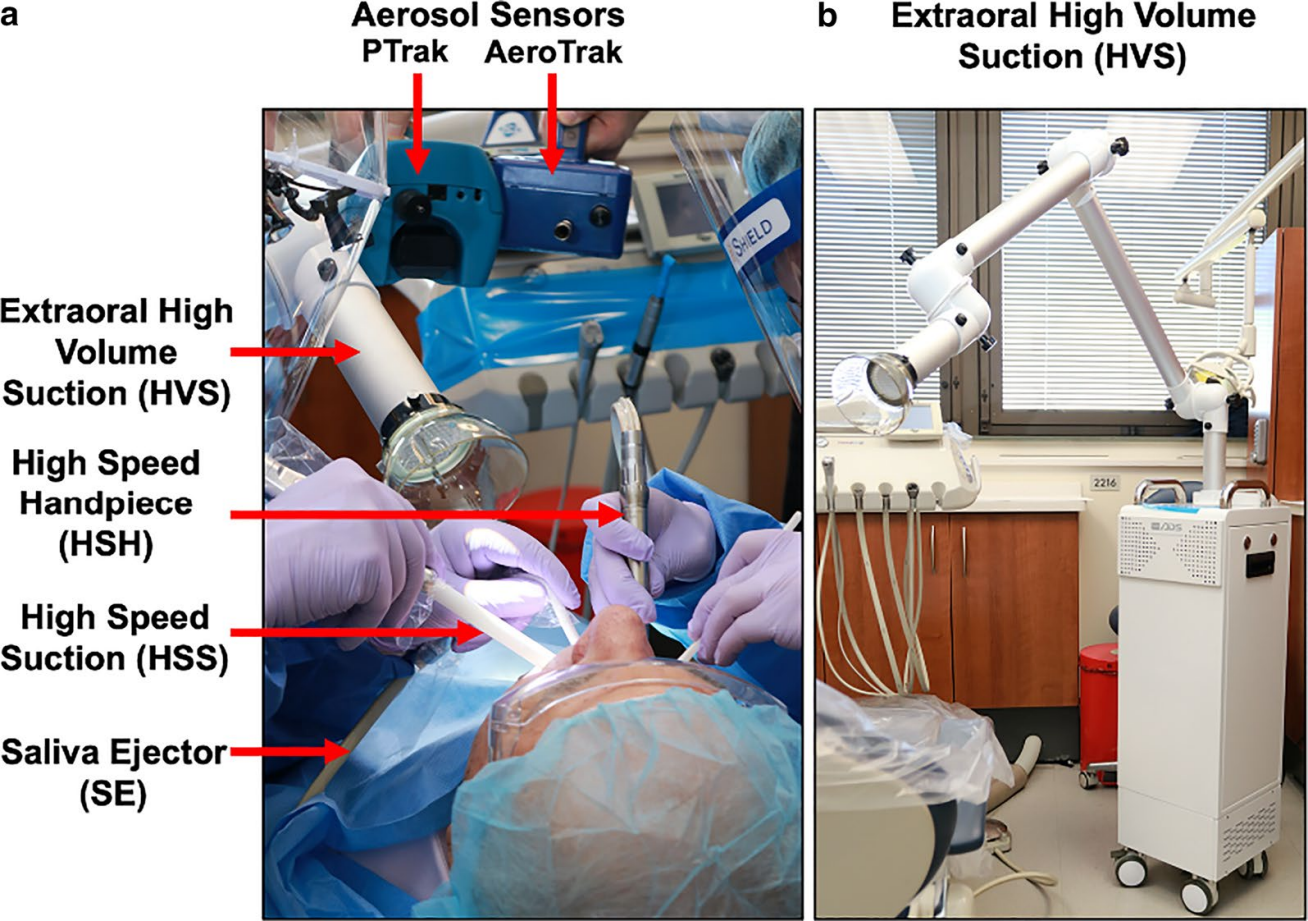
Experimental setup for the dental procedures and aerosol measurements. **a** Locations of the different suction systems including saliva ejector (SE), high speed suction (HSS) and extraoral high volume suction (HVS). Locations of the aerosol meters during data acquisition. **b** The HVS device use for the experiment

**Fig. 2:**
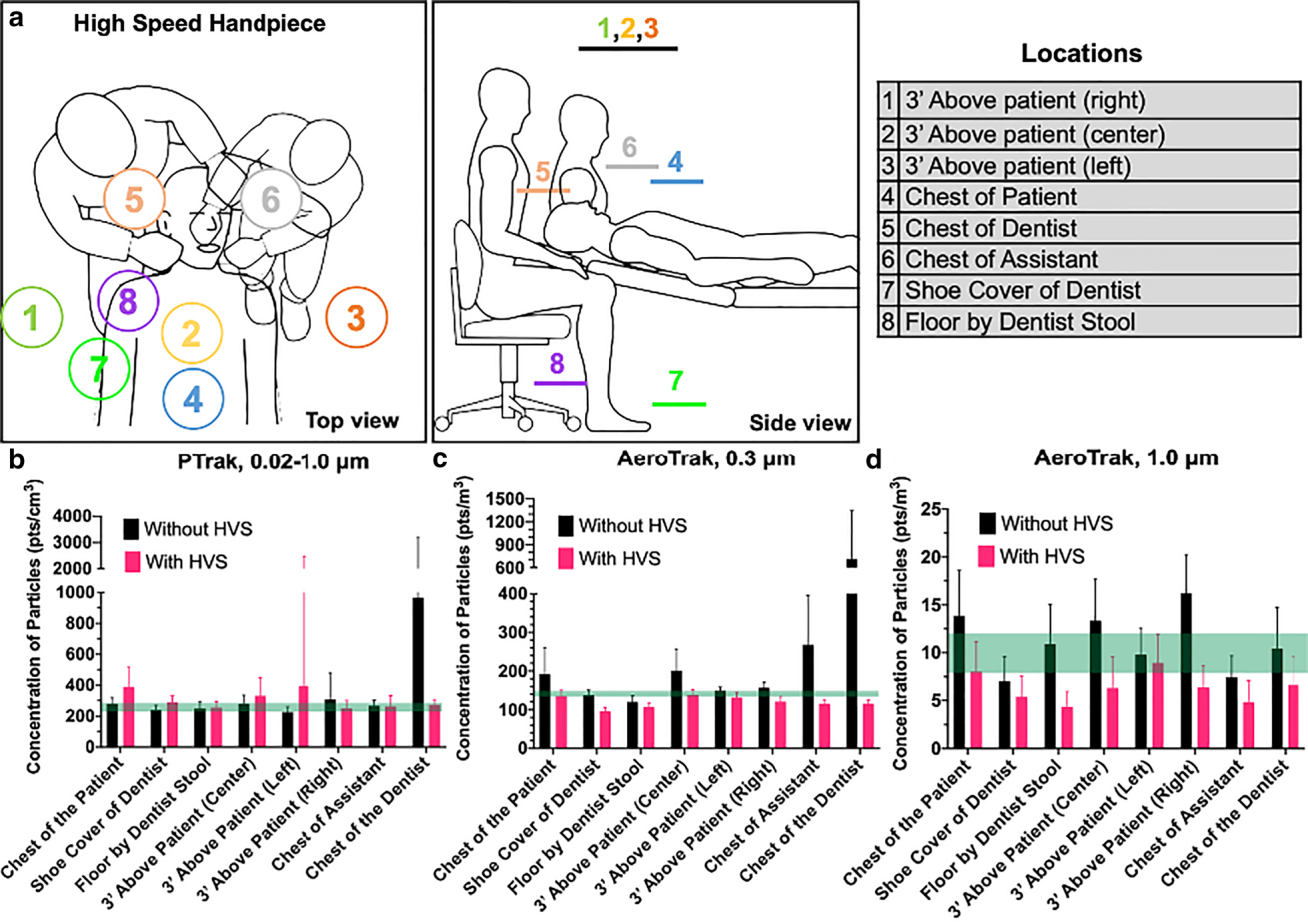
Aerosol spread to different locations in the operatory using HSH + SE + HSS with or without HVS. **a** Positions of dentist, assistant and patient during the mock dental procedure. The scheme corresponds to top and side views. Aerosol level at a total of 8 locations was measured. **b** Concentration of aerosol particles measured at different locations. HSH + SE + HSS without HVS (black bars) and with HVS (pink bars). The green bands correspond to the base line range measured before each experiment

**Fig. 3:**
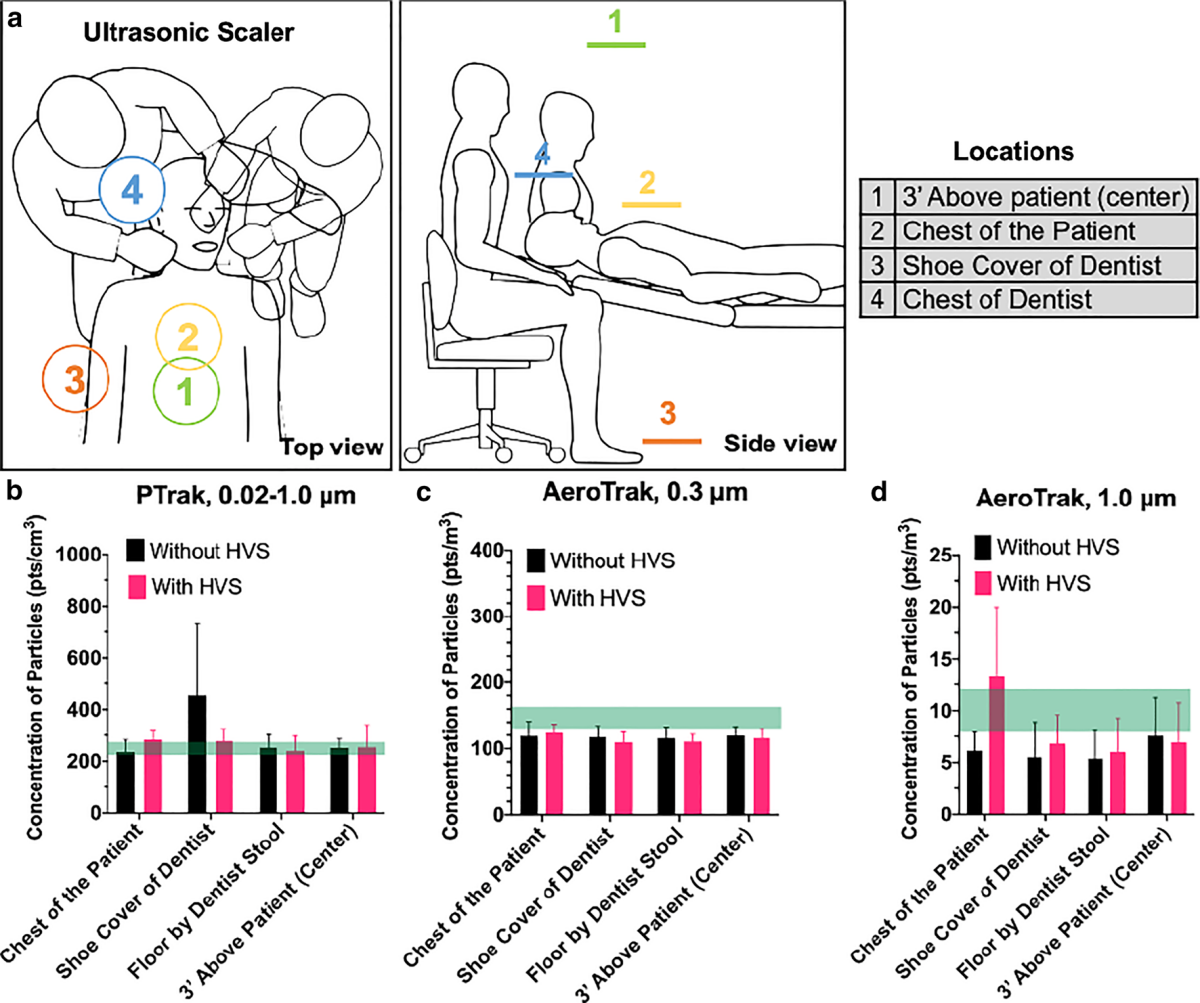
Aerosol generated at different locations in the operatory using an US + SE + HSS with or without HVS. **a** Positions of dentist, assistant, and patient during the dental procedure. The scheme corresponds to top and side views. Aerosol level was measured at total 4 locations. **b** Concentration of aerosol particles measured at different locations. US + SE + HSS without HVS (black bars) and with HVS (pink bars). The green bands correspond to the base line range measured before each experiment

The present study is a proof of concept with one volunteer patient. The experiment was taken place in a dental unit located inside one of the dental clinics at dental school. The dental clinic has multiple operatories divided by modular cabinetry. This clinic, with central conditioning with a temperature of around 70 °F, is regularly used by dental students to provide general dental care. One dentist, one dental assistant, and one volunteer patient participated in mock dental procedures using a HSH (Midwest Stylus, Dentsply). After the completion of procedures with HSH, the patient also received scaling using a Cavitron Plus ultrasonic scaler (Dentsply). The study adhered to relevant guidelines and regulations, and the informed consent was obtained from all participants before procedures. The Temple University Institutional Review Board (IRB) approved the study as exempt, as it was not considered as a human subject research. Pre-operative exam indicated that patient has good oral hygiene in all four quadrants. The patient was seated in a supine position, the dentist was sitting in the 10 to 11-o'clock position and the dental assistant was sitting in the 1 to 2-o'clock position (Figs. 1a, 2a, 3a). HSH with 400,000 rpm and water spray were used to simulate the clinical dental procedure. The handpiece’s head with a mock bur was placed within 1 cm to the teeth surfaces and moved from the upper right to the upper left quadrant, then from the lower left to the lower right quadrant. In each quadrant, the handpiece was moved from the buccal (facial) side to the lingual (palatal) side with constant speed. The whole procedure (four quadrants) was completed within 6 min. In another experiment, the US (medium power setting) was used in a similar manner to scale all maxillary and mandibular teeth. For both experiments (HSH and US), a SE was placed into the patient’s mouth, and a HSS with vented tip was held by the assistant for chairside suction.

To evaluate the effect of using HVS in the control of aerosol spreading, a HVS machine (ADS, Ontario, California, Fig. 1b) was installed on the assistant side and used according to the manufacturers’ instructions. The HVS suction hood was placed 4 inches from the patient’s mouth and the power was set as “7” (maximum is 10). For experiment with HSH, two groups were measured: (1) HSH + SE + HSS and (2) HSH + SE + HSS + HVS. And for the US experiment, two groups were measured: (1) US + SE + HSS and (2) US + SE + HSS + HVS.

### Aerosol measurement

Three aerosol meters (DustTrak 8534, PTrak 8525 and AeroTrak 9306) were used to measure the concentration of particles with size ranges from 0.02 to 10 µm. The DustTrak (DRX photometer) reads the mass of particles in various sized fractions (range of 0.1–10 µm) and their total amount. The DustTrak was kept at a central location within the clinical operatory for 10 min to establish the baseline, then it was kept at the same location during all dental procedures. The results from the DustTrak were expressed as the average mass concentration (mg/m^3^). The PTrak (CPC) reads the minimum, maximum and average values of particle concentration with the size range of 0.02–1.0 µm, and the result was expressed as pts/cm^3^. Data from the AeroTrak (OPC) were obtained in two different channels. The 0.3 µm channel measures particle concentration with sizes smaller than 0.3 µm, and the 1.0 µm channel measures the particles with size ranges from 0.5 to 1 µm. Before the study, the baseline aerosol level was measured as the ambient distribution of particles in the ambient air surrounding the dental chair. During the procedures, measurements were taken at different locations as shown in Figs. 2a, 3a. At each position, it took about 30–45 s to record 10–20 readings. The total time to measure all locations matches the total time for one procedure. For example, in HSH + SE + HSS group, total 8 locations were measured, each location took about 45 s to measure, and the total time for this group is about 6 min.

### Statistical analysis

The mean concentration of particles with varying sizes of the measurements were obtained for the PTrak8525 and AeroTrak 9306. Data from the PTrak correspond to the minimum, maximum and average values of concentration of particles. The AeroTrak measurements were obtained in terms of average and standard deviation. Differences in the concentrations from the devices were compared to baseline readings.

## Results

Before the HSH + SE + HSS procedure was performed, the baseline aerosol level showed an average of 250 pts/cm^3^ (ranged from 247 to 267 pts/cm^3^) from the PTrak; an average of 144 pts/m^3^ (ranged from 104 to 192 pts/m^3^) from AeroTrak 0.3 µm channel, and an average of 10 pts/m^3^ (ranged from 9 to 18 pts/m^3^) from AeroTrack 1 µm channel (Figs. 2 and 3-Green band).

The concentrations of aerosol particles were measured during the procedure using HSH + SE + HSS (Fig. 2b–d). When HSH + SE + HSS was used without HVS (black bars), only the chest of the dentist (967 pts/cm^3^) showed an elevated level of aerosol from the PTrack (Fig. 2b black bars). The chest of the assistant (228 pts/m^3^) and chest of the dentist (712 pts/m^3^) showed an elevated level of aerosol from the AeroTrak 0.3 µm channel (Fig. 2c, black bars). The chest of the patient (14 pts/m^3^), 3 feet above the patient right side (13 pts/m^3^) and 3 feet above the patient center (16 pts/m^3^) showed an elevated level of aerosol from the AeroTrak 1 µm channel (Fig. 2d, black bars). All other locations showed similar aerosol levels compared to the baseline. When the HVS unit was used (Fig. 2-Pink bars), all 8 locations had the aerosol level reduced to similar values to the baseline. The most evident reduction was seen in the chest of the dentist. The aerosol was reduced from 967 to 274 pts/cm^3^ (PTrack), and from 712 to 107 pts/m^3^ (AeroTrack 0.3 channel).

The concentration of aerosol particles during the procedure using the US + SE + HSS were presented in Fig. 3b–d. A total 4 locations (3 feet above the patient, chest of patient, chest of dentist, and shoe cover of dentist) were measured because the trial measurements found that the other sites did not display significant increase in concentration of particles. The results from the PTrack showed that only the shoe cover of the dentist had elevated aerosol levels (455 pts/cm^3^) during the US + SE + HSS procedure (Fig. 3b black bars), while all other locations had aerosol level similar to baseline. When the HVS unit was used, it reduced the aerosol level at the shoe cover of dentist to baseline (Fig. 3b pink bar). The results from the AeroTrak 0.3 µm channel showed that aerosol level at all locations were similar to baseline regardless of the use of HVS or not (Fig. 3c). The results from the AeroTrak 1 µm channel showed that when US + SE + HSS was used without HVS, all locations had aerosol similar or below to baseline level (Fig. 3d black bars). When HVS was used, it increased the aerosol at the chest of the patient (from 6 to 13 pts/m^3^), while it had no effect on other locations (Fig. 3d pink bars).

During both procedures (HSH + SE + HSS, US + SE + HSS), the mass concentrations of aerosol particles (average of 0.012 mg/m^3^) measured by the DustTrak were similar to the baseline (0.011 mg/m^3^), and were not affected by the use of the HVS.

## Discussion

Dental aerosols, mixed with bioaerosols, pose a risk of transmission of SARS-CoV-2 among dental workers and patients. The size of a single COVID-19 virus is 70–90 nm [13], however, the virus does not exist individually but in droplets of > 0.3 µm. Several critical questions need to be addressed: first, how long does aerosol remain in the dental operatory? Studies showed that dental aerosol remains in the operatory 30 min after the dental procedure [14]. Second, how long does SARS-CoV-2 remain vital in aerosol after the dental procedure is completed? A study demonstrated it remains vital in aerosols for at least 3 h, and it was more stable on plastic and stainless steel surfaces than copper and cardboard surfaces [15]. Therefore, the disinfection of the dental operatories, cabinets, and floors must be conducted within several minutes following the completion of dental procedures for each patient. Third, how far does aerosol spread in the dental operatory? Harrel et al. found that an ultrasonic scaler produced aerosols that transmit at least 18 inches from the operative site [3]. Another study found that the maximum contamination was seen in 2 feet away and 1foot above from the site of operation [16]. Fourth, since aerosols may spread to different locations in the dental operatory with different concentrations, it would be interesting to detect the difference among these locations and assess which location(s) exhibit higher concentration of aerosol. In present study, we focused on the fourth question. Veena et al. reported that maximum contamination was found on the right arm of the dentist and left arm of the assistant, in addition to the head, chest and inner surface of the face mask of the dentist and of the assistant [12]. To control dental aerosols, the American Dental Association (ADA) recommended using SE + HSS as a standard in the dental operatory. The present study found SE + HSS had a significant role in the control of aerosol spreading. Only the chest area of the dentist had an elevated level of aerosol, and other 3 locations (3′ above the patient center, 3′ above the patient right and chest of the patient) had slightly elevated levels. Our results support the use of SE + HSS in the dental operatory as recommended by the ADA.

Although several studies intended to assess the dissemination of aerosol by measuring bacterial contamination in the dental operatory [9, 17, 18], only a handful of studies directly measured the aerosol’s dissemination [19–21]. However, these studies either measured the aerosol level in the whole area of dental office which includes multiple dental chairs/operatories, or measured the generation of aerosols in a long period of time (ranged from a day to a week) [20, 21]. In addition, some studies used manikin or extracted teeth instead of patients [12, 19], which failed to simulate dental aerosols that contains a mixture of patient’s saliva and fluid with compressed air and water. To the best of our knowledge, there is no published quantitative evidence on the distribution of size and concentration of aerosols in an individual dental operatory during a specific dental procedure. In the present study, the dentist, assistant, and patient were all positioned in the clinic dental operatory, and the aerosols were tracked and captured in real time while the mock dental procedures were performed. The aerosols were measured by three meters to capture various mass and particle concentrations. This is the first study providing evidence on the generation of different sizes of dental aerosol during a dental procedure.

The highest level of aerosol was found in a triangle area between the chest area of the dentist, of the dental assistant, and of the patient. Current protocol of engineering control, using advanced personal protective equipment (PPE) such as the surgical gown, N95 mask or level 3 surgical mask, eye goggles, face shield, head cover and shoe cover, play a critical role to prevent the spread of pathogenic microorganisms from this area. Dental personnel should strictly follow current guidelines to protect themselves and patients from potential disease transmission. This study also showed that extraoral suction system HVS, as a supplement to SE + HSS, was an effective way to further control of aerosols. HSH + SE + HSS generated the highest level of aerosol in the chest area of the dentist, and HVS was able to reduce it to the baseline level. It was interesting to note that the assistant side had a relatively lower level of aerosol than the dentist's side. This may be due to the fact that both HSS and HVS was approached to patient from the assistant side, and the tilted angle of HSS tip and HVS suction mouth led to a slightly different power of suction.

In the US + SE + HSS group, using HVS increased aerosol at the patient chest area. This was possibly due to the fact that the HVS suction hood was placed further away from the patient’s mouth (more than 4 inches) when this location was measured. The HVS by itself can be a multiplier of aerosol as measured. Exhaust suction provided added velocity to the aerosol stream, and any deflection by the HVS (moved further away from patient mouth) can jettison this stream downward to the patients’ chest area.

Three limitations of the present studies are: (1) when the HSH was used, the bur did not cut the teeth. In clinical scenario, cutting teeth with rotating bur at 400,00 rpm will generate more aerosols. However, compared previous studies that only used a manikin or extracted teeth, the advantage of the present study using a volunteer patient is that we measured the aerosols generated from the human oral cavity, which contains a mixture of patient’s saliva and fluid with compressed air and water. We believe it is a better simulation than those that used a manikin. (2) the present study only measured the aerosol with size smaller than 10 µm. In fact, the aerosol with particle size larger than 10 µm and the splatters (> 50 µm) also contribute to the disease transmission. (3) it is a proof-of-concept study with only 1 volunteer patient. We plan to perform future study with more real patients with actual dental procedures, and measure aerosols with a wider range of particle size.

Finally, to the best of our knowledge, there is no report about COVID-19 infection among dental workers in the dental office since the pandemic started, and most of the transmissions occur due to community interactions and not in institutions following PPE standards. The results of the present study indicated that with SE + HSS, aerosols with size smaller than 10 µm generated in the dental operatory were only at a slightly elevated level compared to baseline, this could partially contribute to the fact that the lack of report about COVID-19 infection among dental workers in a dental office who are strictly following guideline and donning PPE. However, the importance of infection control protocols should not be diminished. In addition to the upgraded PPE, the CDC guidance for dental settings includes preprocedural mouth rinses, and wiping patient’s nostrils and mouth areas with alcohol gauze before dental procedures. Some aerosol generating procedures such as endodontic treatment requires placing an additional rubber dam barrier. The strict compliance with these guidelines will help effectively control aerosol in dental settings. The present study has found that aerosol (with size smaller than 10 µm) generation was minimal for dental procedures relative to the baseline readings. Using the ES + HSS with HVS further reduced aerosol in the dental operatory. This study increases the understanding of the significance of aerosol transmission in the dental operatory and eases the unnecessary levels of anxiety in daily dental practice.

## Conclusions

The increase of aerosol (size smaller than 10 µm) level was minimal during dental procedures when using saliva ejector and high-speed suction. Use of extra-oral high-volume suction further reduced aerosol levels to below baseline level. Accordingly, Temple University granted the school permission to resume its clinical operations under strict PPE conditions, and after 6 months there have been no cases of COVID-19 that are linked to dental care.

## Data Availability

The datasets used and/or analyzed during the current study are available from the corresponding author on reasonable request.
